# Identification of a potential prognostic lncRNA‐miRNA‐mRNA signature in endometrial cancer based on the competing endogenous RNA network

**DOI:** 10.1002/jcb.29200

**Published:** 2019-07-24

**Authors:** Yiwei Wang, Ting Huang, Xiao Sun, Yudong Wang

**Affiliations:** ^1^ Department of Gynecology, International Peace Maternity and Child Health Hospital, School of Medicine Shanghai Jiao Tong University Shanghai China

**Keywords:** ceRNA network, endometrial cancer, lncRNA

## Abstract

Endometrial cancer is one of the most common gynecological malignant tumors. The roles of competing endogenous RNAs (ceRNAs) in this disease, however, remain unclear. In this study, we constructed a ceRNA network to reveal the core ceRNAs in endometrial cancer. Differentially expressed genes were summarized from The Cancer Genome Atlas database, whereupon 140 genes were identified for building the network. Further correlation, survival, and enrichment analyses suggested that these genes may help towards elucidating the molecular mechanisms of endometrial cancer. After validation of the findings with the GSE17025 data set, LINC00958, microRNA‐761, and DOLPP1 were highlighted as the critical genes in the ceRNA network. Our work suggests that LINC00958 may regulate DOLPP1 by “sponging” miR‐761 in endometrial cancer.

## 1 INTRODUCTION

Owing to new technologies such as next‐generation deep sequencing, human genome codes have been shown to be divided into two groups; namely, protein‐coding messenger RNAs (mRNAs), and RNAs without coding potential (also known as noncoding RNAs [ncRNAs]). The high complexity and share of ncRNAs in the genome suggest that they play a significant influence in diseases, including cancer.^1^, ^2^, ^3^, ^4^, ^5^

Among the ncRNAs, the microRNAs (miRNAs, up to 20 nucleotides) and long noncoding RNAs (lncRNAs, more than 200 nucleotides) have been the most focussed upon.^6^, ^7^ miRNAs have been studied extensively with regard to their posttranscriptional repression. They facilitate the degradation and inhibit the translation of target mRNAs through miRNA response elements (MREs).^7^ Meanwhile, studies to date have shown that lncRNAs regulate the patterns of both transcription and posttranscription.^8^ In 2011, Salmena et al were the first to propose the hypothesis of competing endogenous RNAs (ceRNAs), concluding that since various types of RNAs are the targets of miRNAs, any one of them in a pool of targets could competitively interact with its miRNA through the same MRE.^9^ Recent research on solid and hematological malignant tumors uncovered that lncRNAs may function as ceRNAs and could interact with mRNAs by competitively binding with their common miRNAs. Later study on HOTAIR and H19 revealed that ceRNAs could be potential therapeutic targets of cancer.^10^, ^11^, ^12^

Uterine corpus endometrial carcinoma (UCEC) is the fourth most common cancer amongst women worldwide, and its incidence has increased steadily in the past decades.^13^ To date, the roles of ceRNAs in UCEC have not been sufficiently studied. Zhou et al^14^ showed that the lncRNA regulator of reprogramming could act as a ceRNA and “sponge” miR‐145 to inhibit its mediation of endometrial cancer stem cell differentiation. Besides this, Zhang et al^15^ elucidated that lncRNA882 regulated leukemia inhibitory factor by sponging miR‐15b. However, their samples were mainly collected from goat and only a few correlation tests were carried out.^15^

Given the lack of comprehensive investigations on ceRNAs in endometrial cancer, we used available ncRNA and mRNA expression and single‐nucleotide polymorphism array data of UCEC from The Cancer Genome Atlas (TCGA) and constructed a ceRNA‐ceRNA interaction network for endometrial cancer. We were mainly interested in the ceRNAs involved in cancer processes. Finally, one set of ceRNAs (ie, LINC00958, miR‐761, and DOLPP1) was proven to be a potential prognostic signature in endometrial cancer.

## 2 MATERIALS AND METHODS

### 2.1 Data source

**Figure 1:**
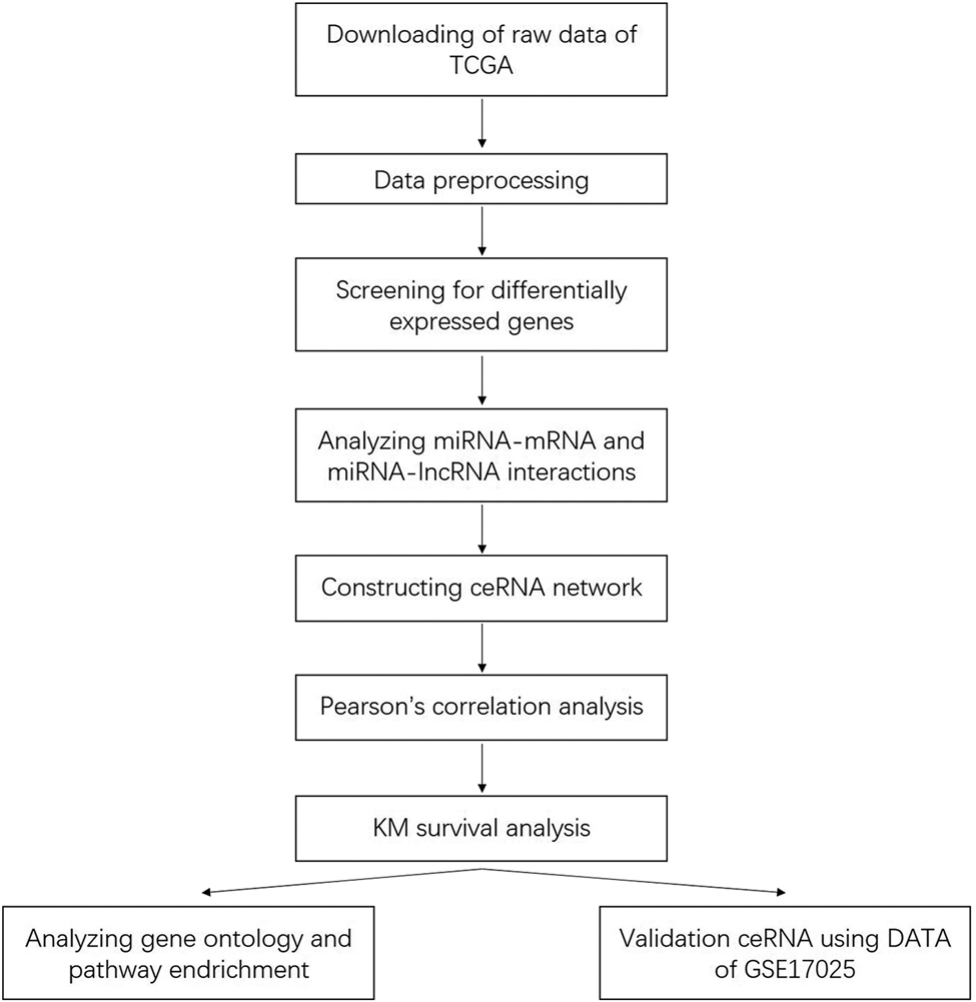
Workflow of the study

The workflow of this study is shown in Figure 1. All raw genomic and clinical data were downloaded from TCGA and the GSE17025 data set, which are free to access. The GSE17025 data set comprised 91 cases of endometrial cancer and 12 control samples. The HTSeq‐Count data of RNA‐Seq and isoform quantification data of miRNA‐Seq were downloaded using the keyword “endometrial cancer.” In total, 571 patients from TCGA were included, with the following sample exclusion criteria: (a) patients with recurrent endometrial cancer and (b) patients who suffered from one or more malignant tumors besides UCEC. In addition, clinical data were also downloaded from TCGA.

### 2.2 Differential expression analysis of UCEC data

To analyze the differential expression of mRNAs (DEmRNAs), lncRNAs (DElncRNAs), and miRNAs (DEmiRNAs) between tumor and normal tissues in UCEC, the bioconductor package GDCRNATools v1.2.0^16^ was used for analyzing the differential expression of genes. A *P*‐value of < .05 and log2|Fold‐change| of > 1 were set up as the criteria.

### 2.3 ceRNA network construction

To identify potential pairs among DEmRNAs, DElncRNAs, and DEmiRNAs in endometrial cancer, data from several databases were retrieved. StarBase v2.0,^17^ miRcode,^18^ and miRTarBase v7.0^19^ were used for identifying the miRNA‐mRNA interactions, whereas StarBase v2.0,^17^ miRcode,^18^ and spongeScan^20^ were used for the miRNA‐lncRNA interactions. UCEC‐specific lncRNAs with an absolute *P*‐value of < .01 were retained. Sponged miRNAs with lncRNAs and mRNAs were identified to construct the ceRNA network, which was then visualized using Cystoscape v3.5.1.

Using the number of target interactions, a hypergeometric test was conducted with the R package GDCRNATools for determining the significance of the shared miRNAs. Regulation similarity and sensitivity correlation were used to measure the regulation pattern. The noneliminated DEmRNA‐DElncRNA pairs were tested by Pearson's correlation analysis with a *P*‐value of < .01 and an *R*‐value of > 0.30.

### 2.4 Survival analysis based on DEmRNAs, DElncRNAs, and DEmiRNAs

Kaplan‐Meier survival analysis was used to evaluate the association between the expression levels of nodes in the ceRNA network and the overall survival of the patients. For Cox regression analysis, data from the Gene Expression Profiling Interactive Analysis (GEPIA) database were retrieved for the pairs satisfying *P < *.05.^21^

### 2.5 Functional annotation of the ceRNA network

We performed Kyoto Encyclopedia of Genes and Genomes (KEGG), Gene Ontology (GO), and Disease Ontology (DO) functional enrichment analyses on the DEmRNAs, DElncRNAs, and DEmiRNAs. KEGG pathways, GO terms, and DO terms with *P* < .05 were significantly enriched.

### 2.6 Statistical analysis

All statistical analyses were performed using SPSS v20.0 (SPSS Inc., Chicago, IL). Differences were considered statistically significant at *P* < .05.

## 3 RESULTS

### 3.1 Identification of DEmRNAs, DElncRNAs, and DEmiRNAs

**Figure 2:**
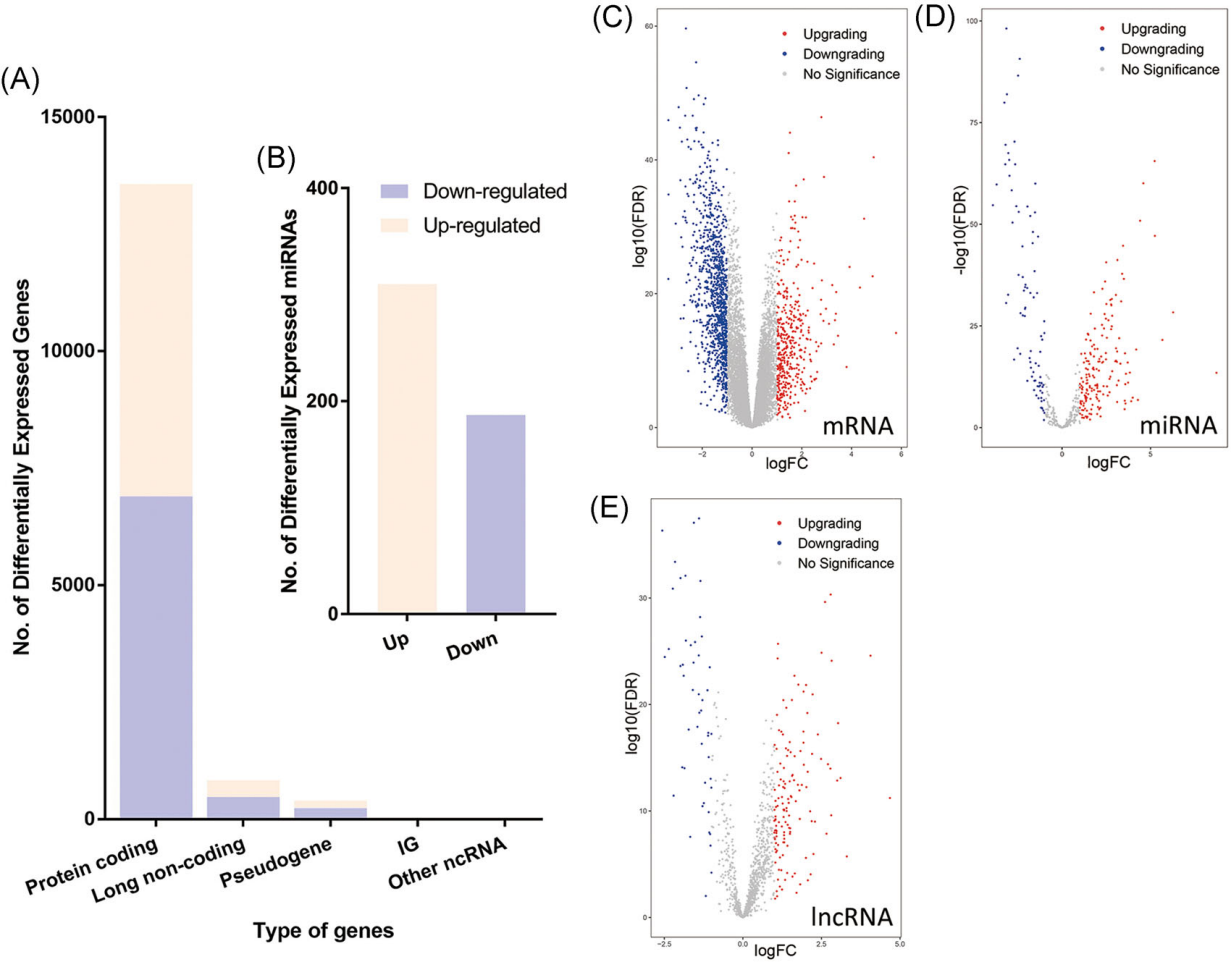
The distributions of differential genes. A, Expression patterns of protein‐coding RNAs, lncRNAs, pseudogenes, immunoglobulin, and other ncRNAs. B, Expression patterns of miRNAs. The ivory represents the upregulated genes and the purple represents the downregulated genes. C‐E, volcano plots of mRNA, miRNA, and lncRNA. The blue plots indicate downregulated genes. The red plots indicate upregulated genes. The gray plots in middle indicate genes that expressed subdifferentially. IG, immunoglobulin; lncRNA, long noncoding RNA; miRNA, microRNA; ncRNA, noncoding RNA

The normalized data of TCGA were annotated as protein‐coding RNAs, lncRNAs, pseudogenes, immunoglobulins, and other ncRNAs. As shown in Figure 2A and 2B, 7532 downregulated genes (6911 mRNAs, 441 lncRNAs, and 180 miRNAs) and 7214 upregulated genes (6617 mRNAs, 294 lncRNAs, and 303 miRNAs) were screened and compared with those of normal tissue. With the thresholds of *P*‐value < .05 and log_2_|Fold‐change| > 1, we detected 1630 DEmRNAs, 51 DElncRNAs, and 88 DEmiRNAs in endometrial cancer compared with the normal controls, and these differentially expressed genes were visualized more intuitively (Figure 2C‐E). The top 10 mRNAs, lncRNAs, and miRNAs exhibiting significant upregulation and downregulation are listed in Table 1.

**Table 1:** Top 10 upregulated and downregulated miRNAs, lncRNAs, and mRNAs in endometrial cancer

Differentially expressed miRNAs			Differentially expressed lncRNAs			Differentially expressed mRNAs		
Gene symbol	*P*‐value	log_2_FC	Gene symbol	*P*‐value	log_2_FC	Gene symbol	*P*‐value	log_2_FC
*Upregulation*			*Upregulation*			*Upregulation*		
*hsa‐miR‐1269a*	9.44E−15	8.73	*PCA3*	9.54E−13	4.68	*DLX1*	7.79E−16	5.79
*hsa‐miR‐205‐5p*	5.55E−30	6.28	*AP006748.1*	7.03E−27	4.06	*NKX2‐3*	8.70E−44	4.89
*hsa‐miR‐4652‐5p*	4.87E−23	5.68	*LINC02170*	6.26E−7	3.31	*ZIC2*	8.96E−25	4.85
*hsa‐miR‐183‐3p*	3.88E−49	5.25	*PCAT14*	9.80E−15	3.10	*DLX2*	6.34E−34	4.50
*hsa‐miR‐183‐5p*	5.75E−68	5.23	*AC009119.1*	3.60E−20	3.03	*SLC45A2*	5.44E−23	4.34
*hsa‐miR‐96‐5p*	2.38E−62	4.60	*AP004608.1*	1.76E−14	3.00	*HOXC6*	2.98E−26	3.92
*hsa‐miR‐182‐5p*	6.54E−53	4.41	*AP000696.1*	5.68E−19	2.83	*TDRD1*	1.90E−10	3.80
*hsa‐miR‐449b‐5p*	7.72E−8	4.29	*AC092535.4*	1.41E−13	2.81	*FOXD1*	2.16E−15	3.46
*hsa‐miR‐4724‐5p*	1.17E−20	4.20	*PCAT7*	7.82E−25	2.80	*MMP26*	2.76E−22	3.38
*hsa‐miR‐891a‐5p*	2.04E−8	4.00	*AC144450.1*	2.55E−10	2.78	*MNX1*	7.12E−19	3.37
*Downregulation*			*Downregulation*			*Downregulation*		
*hsa‐miR‐1‐3p*	7.36E−57	−3.91	*ADAMTS9‐AS1*	1.9E−39	−2.56	*KRT13*	2.34E−24	−3.36
*hsa‐miR‐133a‐3p*	5.30E−62	−3.70	*PGM5‐AS1*	9.94E−27	−2.49	*SERPINA5*	8.82E−40	−3.36
*hsa‐miR‐143‐3p*	1.22E−82	−3.26	*AL049555.1*	1.53E−27	−2.35	*ACTC1*	8.21E−38	−3.35
*hsa‐miR‐139‐3p*	4.47E−67	−3.21	*AC005180.1*	1.47E−33	*CPNE6*	3.85E−33	−3.06	
*hsa‐miR‐100‐5p*	3.97E−72	−3.20	*MIR205HG*	5.60E−13	−2.20	*KCNJ15*	6.67E−52	−2.95
*hsa‐miR‐508‐3p*	2.54E−32	−3.16	*AC005180.2*	2.88E−36	−2.16	*GPX2*	1.28E−48	−2.90
*hsa‐miR‐139‐5p*	1.48E−101	−3.15	*LINC01018*	7.96E−26	−1.99	*IGSF1*	4.04E−40	−2.88
*hsa‐miR‐145‐5p*	8.54E−85	−3.12	*GAS1RR*	1.20E−34	−1.‐3	*SCGB1A1*	4.31E−18	−2.87
*hsa‐miR‐1247‐3p*	2.13E−34	−3.05	*AP000808.1*	8.49E−16	−1.91	*SCGB3A1*	5.89E−31	−2.85
*hsa‐miR‐424‐3p*	5.18E−70	−3.04	*AF165147.1*	5.88E−26	−1.88	*GSTM1*	1.87E−13	−2.83

Abbreviations: lncRNAs, long noncoding RNAs; miRNAs, microRNAs; mRNAs, messenger RNAs.

### 3.2 Construction of the ceRNA network

**Figure 3:**
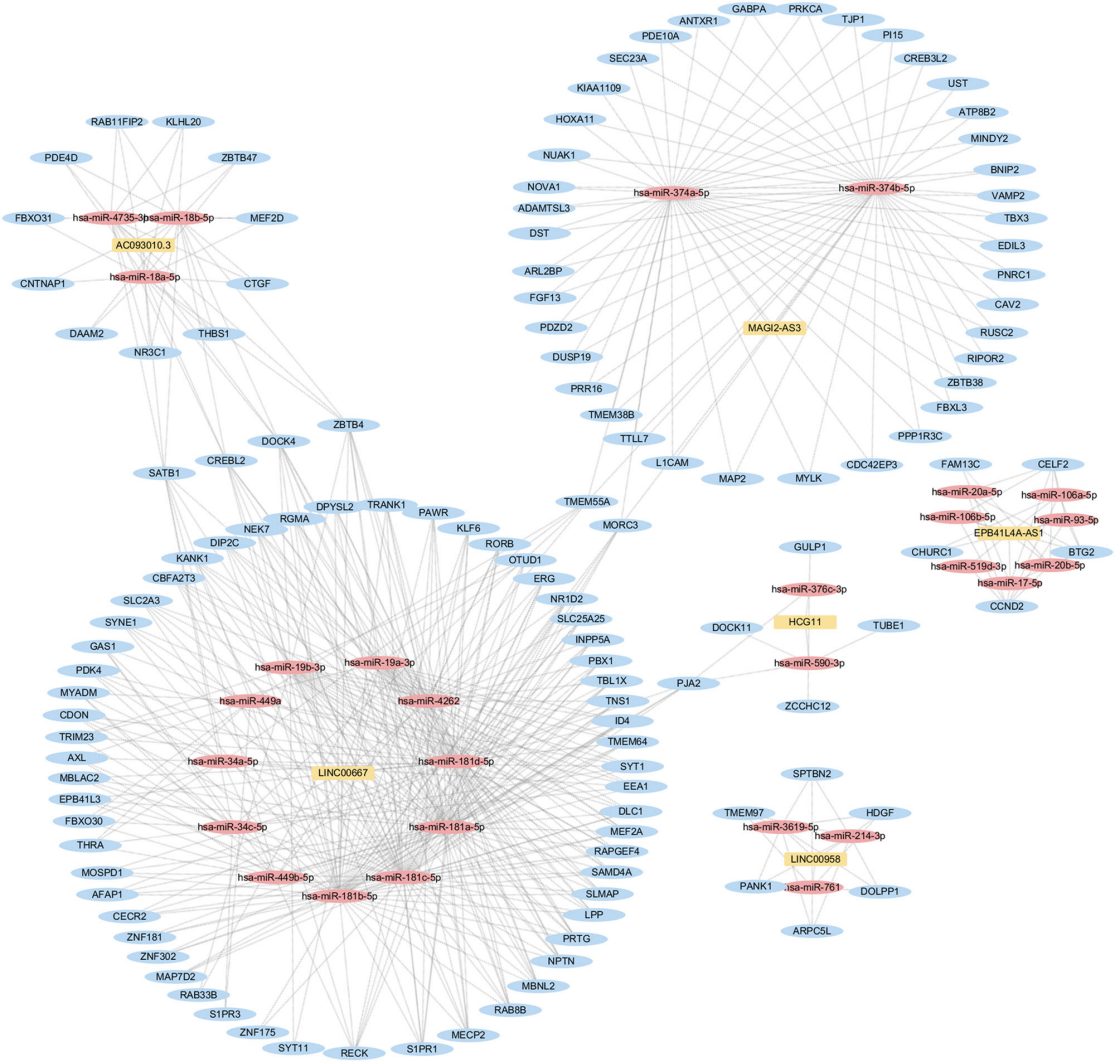
The ceRNA networks. Yellow rectangles represent lncRNAs. Red circles represent miRNAs. Blue circles represent mRNA. ceRNA, competing endogenous RNA; lncRNA, long noncoding RNA; miRNA, microRNA

In total, 1769 differentially expressed genes were selected to build the ceRNA network. We searched potential miRNA‐mRNA and miRNA‐lncRNA binding data from computational prediction and experimental validation databases. In total, we obtained 7812 possible miRNA‐mRNA target pairs and 137 possible miRNA‐lncRNA target pairs. For lower complexity and more reliability, we screened out the isolated DEmiRNAs (target interaction number of > 3) with both the hypergeometric test (*P* < .01) and correlation test (*P* < .01). Consequently, 171 nodes (RNAs) and 520 edges were included in the ceRNA network from the computational prediction (Figure 3). The top four DEmRNAs with rich connections were DOCK4 (nodes = 10), ZBTB4 (nodes = 10), CREBL2 (nodes = 9), and SATB1 (nodes = 9). As shown in Figure 3, six main clusters (lncRNA‐targeted) were grouped, four of which shared the same DEmRNAs. In particular, seven mRNAs (DOCK4, ZBTB4, SATB1, CREBL2, MORC3, TMEM55A, and PJA2) were involved in more than one subnetwork, which suggested that the ceRNA pairs might engage in cross‐talk with other pairs.

### 3.3 Enrichment analyses of the ceRNAs

**Figure 4:**
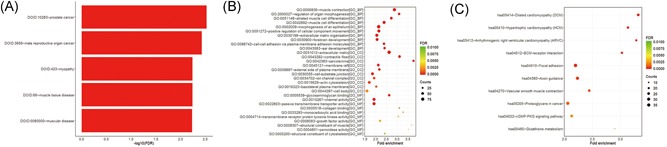
DO enrichment analysis (A), GO enrichment analysis (B), and KEGG pathways (C) of DEmrna. DO, disease ontology; GO, gene ontology; KEGG, Kyoto Encyclopedia of Genes and Genomes; DEmrna, differential expressed mRNA

DO, GO, and KEGG enrichment analyses of the 1769 genes were carried out to further investigate the possible signaling mechanisms of endometrial cancer. Interestingly, the results demonstrated that UCEC has the greatest similarity with prostate cancer, male reproductive organ cancer, and myopathy (Figure 4A). Functional annotation of the ceRNAs revealed that these mRNAs were mainly involved in 821 GO terms, including regulation of organ morphogenesis in the biological process category, sarcolemma in the cellular component category, and structural constituent of muscle in the molecular function category (Figure 4B). In the KEGG analysis, 207 KEGG pathways were enriched, including dilated cardiomyopathy, hypertrophic cardiomyopathy, and arrhythmogenic right ventricular cardiomyopathy (Figure 4C). To the best of our knowledge, these annotated functions have not been studied in endometrial cancer; therefore, our findings provide a rich resource for discovering additional molecular participants in this disease.

### 3.4 Correlation and Kaplan‐Meier analyses of the ceRNAs

**Figure 5:**
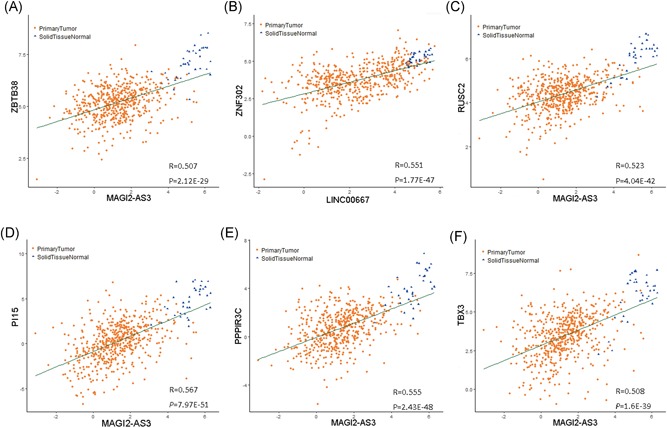
Pearson correlation analysis of paired lncRNAs and mRNAs. A, MAGI2‐AS3 correlates to ZBTB38. B, LINC00667‐ZNF302. C‐F, MAGI2‐AS3 correlates to RUSC2 (C), PI15 (D), PPP1R3C (E), and TBX3 (F). lncRNA, long noncoding RNA; mRNAs, messenger RNAs

Owing to the unique “sponge” function of lncRNAs, it is believed that they should have the same expression patterns as mRNAs. Therefore, 133 DElncRNA‐DEmRNA pairs were tested by Pearson's correlation analysis with *R* ≤ 0.30 and *P*‐value ≥ .05 as exclusion criteria. In particular, six lncRNA‐mRNA pairs with a high *R*‐value were revealed (Figure 5). Among the 58 selected DElncRNA‐DEmRNA pairs, MAGI2AS3‐MYLK (*R* = 0.691), MAGI2AS3‐EDIL3 (*R* = 0.61), and MAGI2AS3‐NOVA1 (*R* = 0.581) were the ones with the highest *R*‐values.

**Figure 6:**
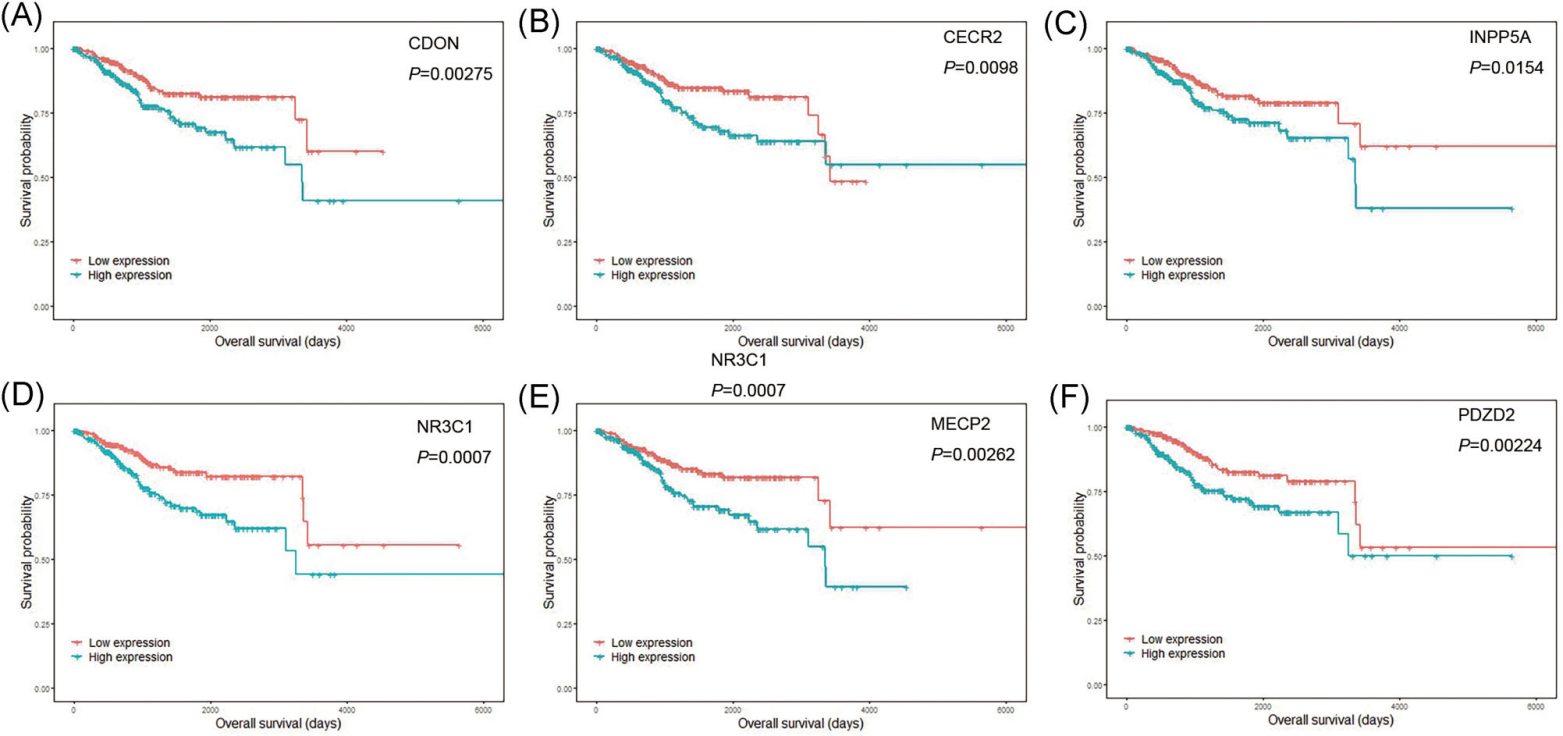
KM survival analysis of DEmrnas. A, CDON. B, CECR2. C, INPP5A. D, NR3C1. E, MECP2. F, PDZD2. Red lines represent patients with lower expression of mRNA. Green lines represent patients with higher expression of mRNA. KM, Kaplan‐Meier; DEmrna, differential expressed mRNA

**Figure 7:**
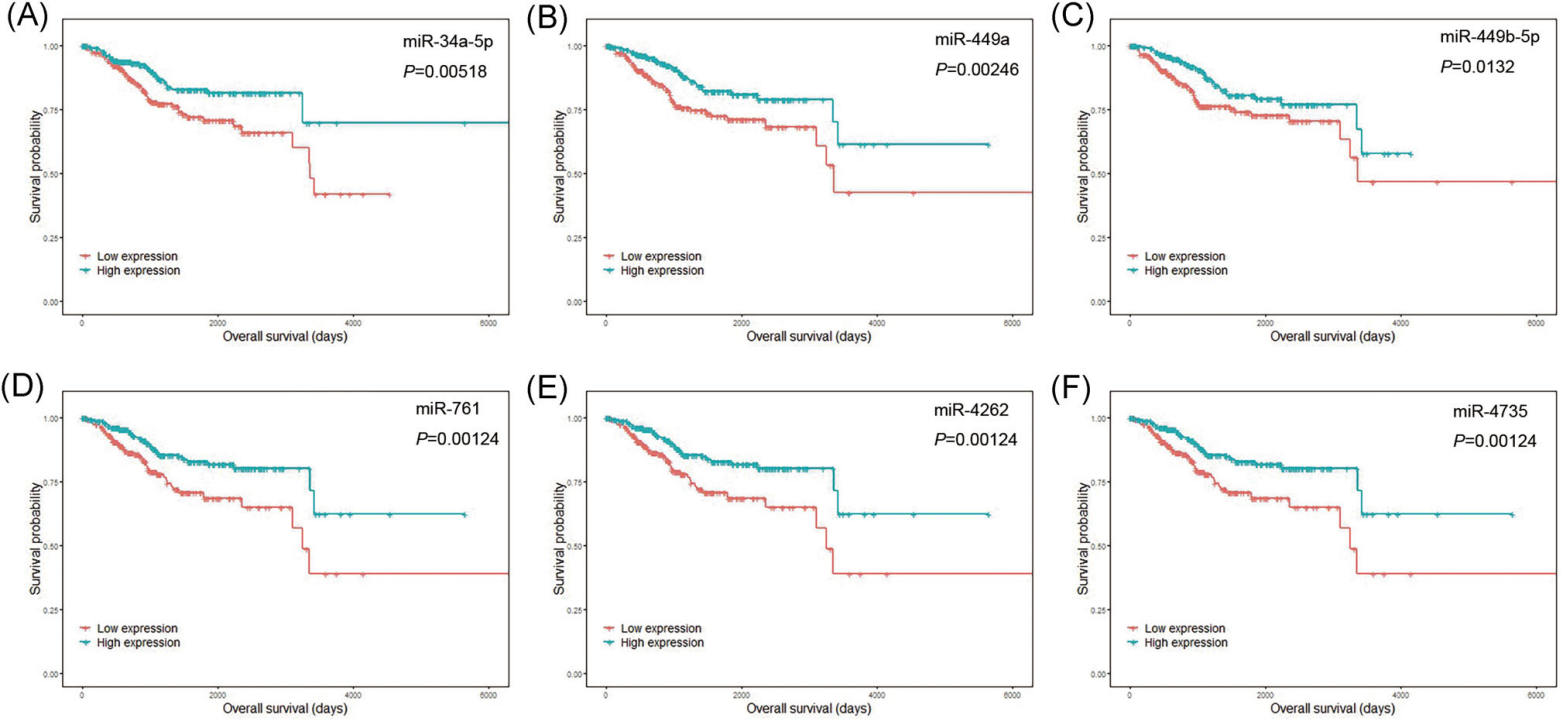
KM survival analysis of DEmirnas. A, miR‐34a‐5p. B, miR‐449a. C, miR‐449b‐5p. D, miR‐761. E, miR‐4262. F, miR‐4735. Red lines represent patients with lower expression of mRNA. Green lines represent patients with higher expression of mRNA. KM, Kaplan‐Meier; DEmirna, differential expressed microRNA

In addition, we conducted an overall survival analysis based on the DEmRNAs and DEmiRNAs to test their potential prognostic value. In total, 23 mRNAs (Figure 6), including NR3C1 (*P* < .0010), UST (*P* = .0014), and MECP2 (*P* = .0026), and seven miRNAs (Figure 7) were obtained with the criteria of *P* < .05. On the basis of their expression patterns and the ceRNA network, ten target pairs were finally highlighted, as displayed in Table 2.

**Table 2:** Validation of selected ceRNAs in GSE17025

mRNA	lncRNA	Regulation pattern	*R*	*P*‐value	Pathological stage expression (*F*‐value)	Pr(>F)
		(*P*‐value)				
NR3C1	AC093010.3	.008^**^	0.2041	.0437*	1.86	0.139
MECP2	LINC00667	.0339*	0.1108	.2984	0.339	0.797
RECK	LINC00667	<.0001^***^	0.3958	<0.0001^***^	0.721	0.541
TMEM55A	LINC00667	.0096^**^	−0.0047	.9647	2.59	0.0543
INPP5A	LINC00667	.9029	−0.0576	.5983	1.3	0.275
DPYSL2	LINC00667	.5068	0.1131	.2883	0.989	0.399
RGMA	LINC00667	.0736	0.1734	.1022	0.0298	0.993
FBXO30	LINC00667	.4400	−0.052	.6260	0.29	0.833
DOLPP1	LINC00958	.0029^**^	0.3161	.0408*	3.81	0.047*
TMEM97	LINC00958	.3356	0.2822	.0071^**^	0.674	0.569

Abbreviations: ceRNAs, competing endogenous RNAs; lncRNA, long noncoding RNA; mRNA, messenger RNA.

* *P* < 0.05.

^**^ *P* < 0.01.

^***^ *P* < 0.001.

### 3.5 Validation of core ceRNAs

To verify the effectiveness and practicability of these ceRNAs, we compared them with another data set, GSE17025 (91 endometrial cancer cases and 12 control cases). As showed in Table 2, the RNA‐Seq results for 10 mRNAs with their related lncRNAs were validated. The expression of three ceRNA pairs (NR3C1‐AC093010.3, RECK‐LINC00667, and DOLPP1‐LINC00958) was proven to be consistent with that in TCGA database. However, we conducted a more rigorous validation of these ceRNAs and found that NR3C1 and RECK failed to be associated with good disease‐free survival. Finally, DOLPP1, LINC00958, and miR‐761 were demonstrated to be the core ceRNAs of endometrial cancer.

## 4 DISCUSSION

Given the yearly increases in the incidence and mortality rate of endometrial cancer, it is of great importance to unravel the molecular mechanisms of this disease.^13^ In our present study, we investigated a potential prognostic ceRNA network of UCEC. To build this lncRNA‐based signature, we processed TCGA data on 571 patients with UCEC and subjected the differentially expressed genes to hypergeometric, correlation, and survival tests. Collectively, our results identified two ceRNAs as being associated with endometrial cancer, which could be used to elucidate the underlying regulatory mechanisms of the disease.

The enrichment analysis results were inconsistent with those of a previous report by Chen et al.^22^ The DO analysis showed that endometrial cancer has the most similarity with prostate cancer, which exceeded our expectations. One conceivable explanation is that luminal progenitor cells may develop from the Müllerian ducts in the same way that the uterus does. Alternatively, numerous genes related to hormones may produce hybrid noise. This finding may explain why GnRHα could display efficacy against both endometrial cancer and prostate cancer.^23^, ^24^ Therefore, mixing the genes of three gynecological cancers together may be not appropriate. Moreover, in the KEGG and GO analyses, several muscle‐related pathways were highlighted owing to the downregulation of potassium calcium‐activated channel subfamily and desmoplakin‐related proteins (data not shown). These findings evident that the regulation of ion channels plays a role in endometrial cancer.

The network provides a straightforward representation of interactions between ceRNAs. The selected lncRNA (LINC00958) and mRNA (DOLPP1) were both expressed at a higher level in endometrial cancer tissue than in healthy tissue samples. Interestingly, a few reports have shown that the silencing of LINC00958 in bladder cancer promotes tumor invasion, which echoes the function of miR‐761 in inducing aggressive phenotypes in breast cancer.^25^, ^26^ As a novel lncRNA and miRNA, LINC00958 and miR‐761 remain basically unknown in tumor biology. The literature has shown that DOLPP1 could mediate N‐glycosylation, and pan‐cancer analysis has indicated that it is upregulated in most tumor types.^27^ These results suggest that further in vivo and in vitro studies are needed.

Collectively, our work has identified the DOLPP1‐LINC00958‐miR‐761 ceRNA set as a good prognostic signature in UCEC. We expect that this systematic analysis of ceRNA interactions will contribute toward our understanding of the molecular pathogenesis of endometrial cancer.

## CONFLICT OF INTERESTS

The authors declare that there are no conflict of interests.
